# β-blockers in septic shock: are we there yet?

**DOI:** 10.5935/0103-507X.20170001

**Published:** 2017

**Authors:** Madalena Coutinho Cruz, Luís Reis

**Affiliations:** 1 Departamento de Cardiologia, Hospital de Santa Marta, Centro Hospitalar de Lisboa Central - Lisboa, Portugal.; 2 Unidade de Urgência Médica, Hospital de São José, Centro Hospitalar de Lisboa Central - Lisboa, Portugal.

## Background

Septic shock is characterized by circulatory collapse and diminished tissue perfusion, leading to organ dysfunction in the setting of systemic infection. The mechanisms of septic shock are incompletely understood; moreover, its incidence is increasing, and its mortality remains unacceptably high. In addition to antimicrobial and supportive treatments, no other therapy has a survival benefit, despite multiple attempts at developing one.^(1)^ With the article by Morelli et al.,^(2)^ a new hope has emerged.

## Septic shock and the adrenergic system

The adrenergic system plays a key role in modulating cardiovascular, immune, hemostatic and metabolic functions. It is upregulated in septic shock through the activation of different adrenoreceptors that have distinct and sometimes opposite effects.^(3)^ The adrenergic system serves as an initial adaptive response to maintain homeostasis by elevating heart rate, stroke volume and mean arterial pressure, keeping the balance of inflammation and coagulation and providing sufficient nutrients to cells. However, over the long term, the high output of endogenous catecholamines causes an imbalance in this regulatory function and perpetuates organ dysfunction.^(4)^ To make matters worse, there is also an adrenergic storm caused by the use of vasopressor therapy, which is the mainstay of supportive treatment for fluid-unresponsive septic shock.^(1)^ Noradrenaline, adrenaline and dopamine are used for their α-adrenergic vasoconstrictor effects, but they also act upon β-adrenergic receptors, mainly β1.

As such, they can promote tachydysrhythmias and cardiomyopathy and upregulate inflammatory and coagulation pathways,^(3,4)^ which is deleterious.

## Sepsis-induced cardiomyopathy

A particular occurrence in septic shock is the development of cardiac dysfunction. The definition of sepsis-induced cardiomyopathy is not established, but it is historically characterized by reduced left ventricular (LV) ejection fraction, LV dilation and complete recovery in 7 to 10 days.^(5)^ Right ventricular (RV) dysfunction and dilation are also observed.^(6)^ Its cause is not completely understood, but inflammatory mediators and adrenergic hyperstimulation are important contributors to impairing myocyte signaling transduction and reducing cardiac contractility. Tachycardia, which is common in septic shock and is a known predictor of poor prognosis, promotes cardiac dysfunction by increasing oxygen requirements and diminishing diastolic cardiac filling and coronary perfusion. An estimated 50% of septic shock patients develop cardiomyopathy, as assessed by echocardiography.^(5)^ However, considering that the LV ejection fraction is dependent not only on LV contractility but also on pre- and afterload (that are in turn related to the quantity of fluids and vasopressors imposed on each patient), cardiac dysfunction would likely be present in virtually all patients with septic shock if they were evaluated using a method less dependent on the degree of resuscitation. Contrary to previous beliefs, there is currently no clinical evidence that links LV or RV systolic dysfunction or dilation to prognosis in septic shock.^(6)^ Nevertheless, a recent meta-analysis reported that diastolic dysfunction, which may be more common, is related to mortality.^(7)^

## Septic shock and β-blockers

There has been a growing interest in the use of β-blockers in septic shock. It was hypothesized that the administration of β1-selective blockers could protect patients from the toxicity of endogenous and exogenous catecholamines and ameliorate cardiac function and the homeostasis of immunologic and coagulation processes. These effects have already been proven in animal models. However, the results for prognosis have not been consistent,^(3)^ and some concerns about the danger of precipitously reducing cardiac output and blood pressure remained.

The first randomized clinical trial (RCT) conducted to assess the effect of β-blockade on tachycardia and other hemodynamic parameters in septic shock enrolled 154 patients who remained tachycardic (heart rate (HR) > 94 beats per minute (bpm)) and on a noradrenaline infusion after 24 hours of standard resuscitation. Half of them were randomized to receive an esmolol infusion, and all patients in this group achieved the target HR (80 - 94 bpm) with no adverse effects on systemic or pulmonary hemodynamics. In fact, stroke volume increased, which suggests an optimization of cardiac efficiency and myocardial oxygen utilization. There was also an improvement in perfusion markers, such as arterial lactate and pH, oxygen consumption and estimated glomerular filtration rate. Fluid and vasopressor requirements were decreased, and, most importantly, there was a significantly lower 28-day mortality in the patients who received esmolol.^(2)^

While systemic hemodynamic parameters are accessible for monitoring patients with circulatory collapse, the cornerstone of organ dysfunction in septic shock is the microcirculation, which is less easily assessed in everyday clinical practice.^(8)^ The same authors published a pilot study that added the evaluation of the sublingual microcirculation to a similar design. They noted an improvement in two parameters (blood flow and heterogeneity index), whereas the other two (De Baker score and perfused vessel density) were unchanged in the group receiving esmolol. While it is safe to conclude that β-blockade poses no danger to the microcirculation, it is not possible to assume that there was an improvement in this vascular bed.^(9)^

## Limitations of the current randomized clinical trial

Although it is clear that there is a theoretical rationale for using selective β-blockers that is supported by animal studies, the clinical evidence is still scarce, and many questions remain to be answered.

The RCT by Morelli et al. showed a significant survival benefit (albeit no independent effect of esmolol on mortality was found in the multivariate analysis), but the trial was not designed for this purpose, and the mortality was extremely high in the control group.^(2)^ This raises the concern that there might be a bias in patient selection, in which the persistence of tachycardia after 24 hours of resuscitation is related to worse prognosis. It is not known whether less severe patients would derive a similar benefit. Additionally, half of the patients received the inodilator levosimendan (that improves diastolic function), and its influence on outcome is unknown. The other studies that address the influence of β-blockers on mortality either show a neutral effect or are flawed in their design and preclude the extrapolation of the results to the general population.^(10)^

Another issue is the fact that most studies published to date rely on reducing tachycardia to obtain an improvement in cardiac function.^(4)^ It is unknown whether the mortality benefit is produced by the treatment of tachycardia itself or by its effect on cardiac function. Because there is no relationship between systolic dysfunction and prognosis,^(6)^ it is possible that the improvement in stroke volume is not solely responsible for the mortality benefits. However, it is reasonable to hypothesize that the reduction in heart rate would have beneficial effects on diastolic function, as well by augmenting diastolic filling time, and could potentially confer an improvement in prognosis.^(7)^ Unfortunately, this is only speculative, as no diastolic parameters were assessed. Additionally, no study assessed the optimal heart rate in septic shock, as the targets for its reduction were arbitrarily set.^(2,9,10)^

An additional matter to be discussed is the fact that sinus tachycardia is often a response to an insult, such as fever, anxiety, pain, anemia, hypoxemia, thyrotoxicosis, electrolyte and acid-base abnormalities.^(11)^ These factors should be frequently monitored and thoroughly addressed before true deleterious tachycardia, caused solely by hyperadrenergic status, is treated with β-blockers. The role of heart rate in guiding the management of critically ill patients would also be lost if the use of β-blockers in septic shock were to become widespread. In particular, volume status/fluid responsiveness and autonomic nervous system function are important issues in intensive care patients. Nevertheless, static hemodynamic measurements are presently being replaced with dynamic ones for the assessment of volume status/fluid responsiveness,^(12)^ and a number of methods for autonomic nervous system evaluation beyond those dependent on heart rate are being developed.^(13)^ Nevertheless, it would be useful to have a clear view of how to monitor these patients.

Furthermore, only one non-randomized prospective trial evaluated the effect of β-blockers on microcirculation, and the results were not conclusive.^(9)^ Because microcirculation appears to play a more important role than macrocirculation in sepsis,^(8)^ it would be essential to understand the action of β-blockers on the microvascular bed.

Lastly, given the ubiquitous distribution of the adrenergic system, it cannot be excluded that β-blockers could also exert their influence on outcome through their non-cardiac anti-inflammatory and anticoagulation effects.

## Conclusion

The potential benefits of β-blockers in septic shock patients are vast and include the amelioration of cardiac function and microcirculation, anti-inflammatory and anticoagulation effects, and survival benefits. However, although very promising, there is currently not enough evidence to advise the use of β-blockers in everyday practice, and there is an urgent need for more studies. Two large RCTs (ESMOSEPSIS - Esmolol Effects on Heart and Inflammation in Septic Shock and THANE - Hemodynamic Tolerance and Anti-inflammatory Effects of Esmolol During the Treatment of Septic Shock) are presently recruiting and should provide new insight into the effects of β-blockers on systemic hemodynamics, including diastolic function and microcirculation, as well as the immune system and mortality; hopefully, these RCTs will provide a better understanding of the selection process of the ideal patients for this therapy and how to monitor them.
